# Ongoing Spillover of Hantaan and Gou Hantaviruses from Rodents Is Associated with Hemorrhagic Fever with Renal Syndrome (HFRS) in China

**DOI:** 10.1371/journal.pntd.0002484

**Published:** 2013-10-17

**Authors:** Wen Wang, Miao-Ruo Wang, Xian-Dan Lin, Wen-Ping Guo, Ming-Hui Li, Sheng-Hua Mei, Zhao-Mei Li, Mei-Li Cong, Rui-Lan Jiang, Run-Hong Zhou, Edward C. Holmes, Alexander Plyusnin, Yong-Zhen Zhang

**Affiliations:** 1 State Key Laboratory for Infectious Disease Prevention and Control, Collaborative Innovation Center for Diagnosis and Treatment of Infectious Diseases, National Institute for Communicable Disease Control and Prevention, Chinese Center for Disease Control and Prevention, Beijing, China; 2 Longquan Center for Disease Control and Prevention, Longquan, Zhejiang province, China; 3 Wenzhou Center for Disease Control and Prevention, Wenzhou, Zhejiang province, China; 4 Department of Inner Medicine of Longquan Hospital, Longquan, Zhejiang Province, China; 5 Sydney Emerging Infections and Biosecurity Institute, School of Biological Sciences and Sydney Medical School, The University of Sydney, Sydney, New South Wales, Australia; 6 Department of Virology, Infection Biology Research Program, Haartman Institute, University of Helsinki, Helsinki, Finland; Swiss Tropical and Public Health Institute, Switzerland

## Abstract

**Background:**

Longquan City, Zhejiang province, China, has been seriously affected by hemorrhagic fever with renal syndrome (HFRS) since the first cases were registered in 1974. To understand the epidemiology and emergence of HFRS in Longquan, which may be indicative of large parts of rural China, we studied long-term incidence patterns and performed a molecular epidemiological investigation of the causative hantaviruses in human and rodent populations.

**Method/Principal Findings:**

During 1974–2011, 1866 cases of HFRS were recorded in Longquan, including 20 deaths. In 2011, the incidence of HFRS remained high, with 19.61 cases/100,000 population, despite the onset of vaccination in 1997. During 1974–1998, HFRS cases in Longquan occurred mainly in winter, while in the past decade the peak of HFRS has shifted to the spring. Notably, the concurrent prevalence of rodent-borne hantaviruses in the region was also high. Phylogenetic analyses of viral sequences recovered from rodents in Longquan revealed the presence of novel genetic variants of Gou virus (GOUV) in *Rattus* sp. rats and Hantaan virus (HTNV) in the stripe field mice, respectively. Strikingly, viral sequences sampled from infected humans were very closely related to those from rodents.

**Conclusions/Significance:**

HFRS represents an important public health problem in Longquan even after years of preventive measures. Our data suggest that continual spillover of the novel genetic variant of GOUV and the new genetic lineage of HTNV are responsible for the high prevalence of HFRS in humans. In addition, this is the first report of GOUV associated with human HFRS cases, and our data suggest that GOUV is now the major cause of HFRS in this region.

## Author Summary

Hemorrhagic fever with renal syndrome (HFRS) is a major public health problem in China despite human vaccination. We investigated the epidemiology and emergence of HFRS in Longquan (Zhejiang Province), a rural area with a high incidence of HFRS. During 1974–2011, a total of 1866 cases of HFRS were recorded in Longquan, including 20 deaths. Strikingly, phylogenetic analyses of viral sequences sampled from local rodents in Longquan revealed the presence of novel variants of Gou virus (GOUV) in *Rattus* sp. rats and Hantaan virus (HTNV) in the stripe field mice, respectively. Moreover, viral sequences sampled from infected humans in Longquan were very closely related to those from rodents. Overall, these data indicate that there is a continual spillover GOUV and HTNV from rodents to humans in Longquan, and this might be responsible for the high prevalence of HFRS. As well as highlighting the importance of the human-animal interface, these data also suggest that GOUV is now the major cause of HFRS in this region.

## Introduction

Hantaviruses are important zoonotic pathogens. Although they can establish a persistent and asymptomatic infection in their natural rodent reservoirs [1], in humans hantaviruses can cause two severe diseases: hemorrhagic fever with renal syndrome (HFRS) and hantavirus (cardio) pulmonary syndrome (HPS) [2]. In Eurasia HFRS is associated with Hantaan virus (HTNV), Seoul virus (SEOV), Amur/Soochong virus (ASV), Dobrava-Belgrade virus (DOBV), Saaremaa virus (SAAV), Sochi virus, and Puumala virus (PUUV), whereas HPS is due to the infection of Sin Nombre virus (SNV), Andes virus (ANDV), and other viruses in the Americas [2], [3]. The clinical severity of HFRS is related to the etiologic agents involved [4]–[8], with DOBV and HTNV being the most dangerous representatives, with fatality rates of up to 15% [4]–[7]. In contrast, SEOV usually causes a milder form of HFRS with a mortality rate of approximately 1% [6], [7]. PUUV causes a mild disease referred to as nephropathia epidemica (NE) with a mortality rate ranging from 0.1% to 0.3% in Europe 5,8. HFRS cases caused by HTNV mainly occur in the winter, while the HFRS cases caused by SEOV peak in the spring and summer [9], and which likely reflects occupation-connected differences in exposure to rodents in different seasons.

Following the implementation of comprehensive preventive measures and socioeconomic development, the numbers of HFRS cases and fatalities in China have decreased dramatically, although remain the highest globally [7]. In China, the most prevalent hantaviruses are HTNV and SEOV carried, respectively, by the striped field mouse (*Apodemus agrarius*) and Norway (or brown) rat (*Rattus norvegicus*) [6], [7], [9], [10]. To date, only these two viruses have been identified to cause HFRS in China. However, hantaviruses from bats, insectivores, and rodents (e.g. Dabieshan virus (DBSV), Gou virus (GOUV), Longquan virus (LQUV), Thottapalayam virus (TPMV)) have also been documented [7], [11]–[15], although whether they are associated with human disease is unclear.

Longquan is a county-level city located in the southwestern part of Zhejiang Province. It includes both urban and rural areas, with a population of approximately 280,000. More than 90% of the Longquan's total area is mountainous. In 1974, the first HFRS case was recorded in Longquan. Since that time, Longquan has been one of the most severely affected regions in Zhejiang and in China as a whole. However, little is known about the epidemiology and etiologic agents of HFRS in this region. Our recent surveys in Longquan revealed at least nine species of rodents and insectivores, with *A. agrarius* and *R. norvegicus* dominant in rural and residential areas, respectively [16]. Herein we report the changing incidence of HFRS in Longquan, the genetic characterization of the etiologic agents (hantaviruses) circulating in local rodents, and their connection to the human population.

## Materials and Methods

### Ethics statement

This study was reviewed and approved by the ethics committee of National Institute for Communicable Disease Control and Prevention, Chinese Center for Disease Control and Prevention (Chinese CDC). All animals were treated in strict according to the guidelines for Laboratory Animal Use and Care from the Chinese CDC and the Rules for the Medical Laboratory Animal (1998) from the Ministry of Health, China. These protocols were approved by the National Institute for Communicable Disease Control and Prevention of the China CDC. All surgery was performed under ether anesthesia, and all efforts were made to minimize suffering. Collecting human serum samples from HFRS patients was also approved by the ethics committee of National Institute of Communicable Disease Control and Prevention of the China CDC, according to the medical research regulations of Ministry of Health, China. A signed individual written informed consent was obtained from each of five patients when their blood samples were collected.

### Collection of epidemiological data on HFRS

Records for HFRS cases occurring during 1974–2011 were obtained from the Longquan Center for Disease Control and Prevention. Until 1982, HFRS cases were defined according to the national standard of clinical criteria, and confirmed by detection of hantavirus-specific IgM and IgG antibodies against HTNV or SEOV. From 1982 clinical cases were confirmed by a four-fold or greater titer increase of IgG antibodies in paired sera, as well as a IgM antibody titer >1∶20 in single serum as scored positive by an indirect immunofluorescent assay (IFA) (see below) [9]. The reaction pattern of positive serum was characterized as scattered and green granular cytoplasmic fluorescence in hantavirus-infected Vero E6 cells. The incidence rates of HFRS during 1974–2011 were calculated according to the population census number for each year.

### Animal samples

Small mammals were trapped in fields and residential areas in Longquan during 2008–2011. Cages with a treadle release mechanism were used for live trapping according to the protocols described previously [17]. Traps were set in the same fields or residential areas during each season. Lung and kidney samples were collected from the trapped animals and stored in liquid nitrogen. All surgery was performed under ether anesthesia to reduce suffering. Ethanol-cleaned instruments were used for each animal.

### Detection of hantavirus antibody or antigen

Serum samples collected from five patients who suffered from acute HFRS during 2009–2011 were also studied. These serum samples were tested by IFA using HTNV-infected or GOUV-infected Vero-E6 cells as antigens [18]. The secondary antibody used was fluorescei-isothiocyanate-conjugated goat anti-human IgG or IgM (Southern Biotech, Birmingham, Alabama, USA).

Hantavirus antigen in lung or kidney tissues from rodents and insectivores was detected by IFA as described previously [18], with rabbit antibodies against the mixed antigens of HTNV/76-118 and SEOV/L99 prepared in this laboratory as the primary antibodies and FITC-labeled goat anti-rabbit IgG antibodies used as the secondary antibodies (Sigma, St. Louis, MO, US). Generally, lung tissues were tested first, and kidney tissues were tested if lung tissues were found to be negative.

### RT-PCR and sequencing

Total RNA was extracted from hantavirus antigen-positive lung and kidney tissues, and human serum samples, using the TRIzol reagent (Invitrogen, San Diego, CA) according to the manufacturer's instructions. cDNA of the Small (S) and Medium (M) segments of the hantavirus genome was prepared with AMV transcriptase (Promega, Beijing, China) in the presence of primer P14 [19]. Partial or complete sequences of the S and the M segments were amplified as described previously [10], [20], [21]. All voles and insectivores were also screened for hantaviruses using RT-PCR as described previously [22].

DNA products were purified by a QIAquick Gel Extraction kit (QIAGEN, Beijing, China) and subjected to direct sequencing using the ABI-PRISM Dye Termination Cycle Sequencing ready reaction kit and a ABI-PRISM3730 genetic analyzer (Applied Biosystems, Carlsbad, CA, USA).

### Phylogenetic analysis

The genome sequences of hantaviruses were aligned using the ClustalW method implemented in the Lasergene program, version 5 (DNASTAR, Inc., Madison, WI). Nucleotide (nt) and amino acid (aa) sequence similarities were calculated using DNAStar. Phylogenetic trees for each segment were inferred using the Bayesian method implemented in MrBayes 3.1 [23] and the Maximum likelihood (ML) method available in the RAxML Blackbox webserver [24], employing the best-fit GTR+I+Γ model of nucleotide substitution as determined using jModeltest [25]. Trees were visualized with the TreeView software [26].

### Accession numbers

The GenBank accession numbers for the sequences obtained here are JQ912697 to JQ912907, and KC344236 to KC344269 (Table S2).

## Results

### Prevalence of HFRS in Longquan, China

**Figure 1:**
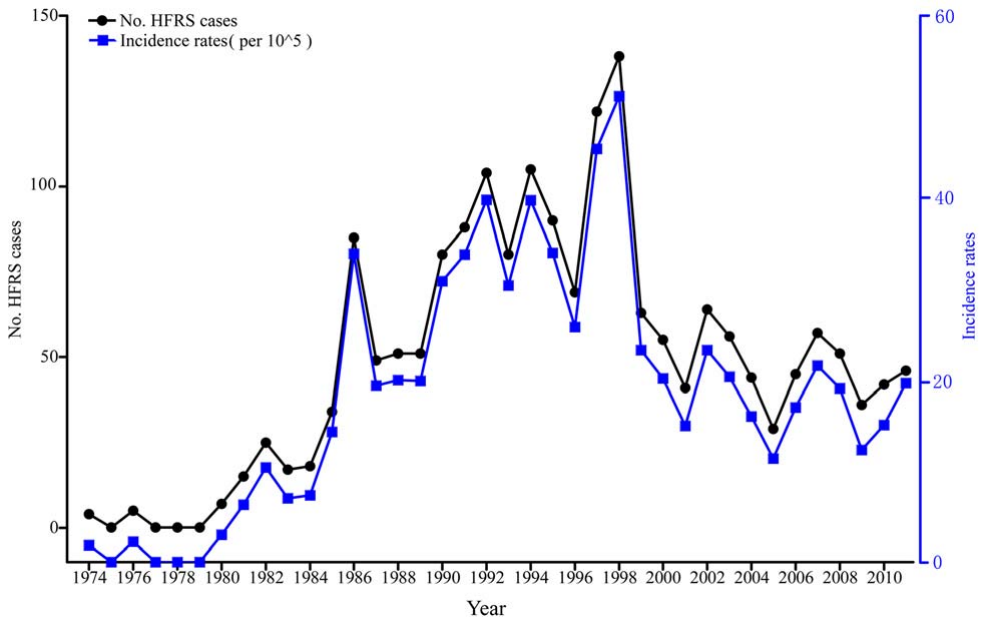
Incidence of HFRS in Longquan. Annual numbers and incidence (cases/100,000 population) of HFRS reported during 1974–2011 in Longquan city, Zhejiang Province, China.

The first clinical HFRS case in Longquan was reported in 1974 (Figure 1). During the 38-year period between 1974 and 2011, a total of 1,866 HFRS cases were registered in this city. Only nine cases were recorded in the 1970s, such that the annual incidence of HFRS increased dramatically during 1980s and 1990s. A peak of 138 cases (51.2 cases/100,000 population) was reached in 1998, after which it decreased, likely in part due to the onset of hantavirus vaccination in 1997 and the intense rodent control efforts undertaken in China [7]. In total, more than 63,000 people have been vaccinated either by inactivated vaccines (Youerjian, Tianyuan Bio-Pharma, Hangzhou, China) for HTNV (during 1997–2000) or purified bivalent vaccine for HTNV and SEOV cultured in sand rat renal cells (Youerjian, Tianyuan Bio-Pharma, Hangzhou, China) or Vero cells (Royal, Royal (Wuxi) Bio- Pharmaceutical, Wuxi, China) (during 2001–2011). However, despite this vaccination the incidence of HFRS remained relatively high during 1999–2011, with between 11.15 and 23.6 cases/100,000 population.

During 1974–2011, a total of 20 patients died of HFRS in Longquan, with an average fatality rate of 1.07%. The highest fatality rates were observed during the first 10 year period (1974–1983), and reached 11% (10 fatal cases of 91 cases). Notably, all fatal cases occurred in autumn and winter. Additional fatalities were recorded in 1985 (1), 1986 (2), 1989 (1), 1992 (1), 1997 (1), 1998 (1), 2002 (1), and 2006 (2): these cases occurred in autumn and winter, with the exception of one death in March 2002. No patients have died of HFRS since 2007, likely reflecting improvements in disease treatment.

### Seasonal distribution of HFRS cases

**Figure 2:**
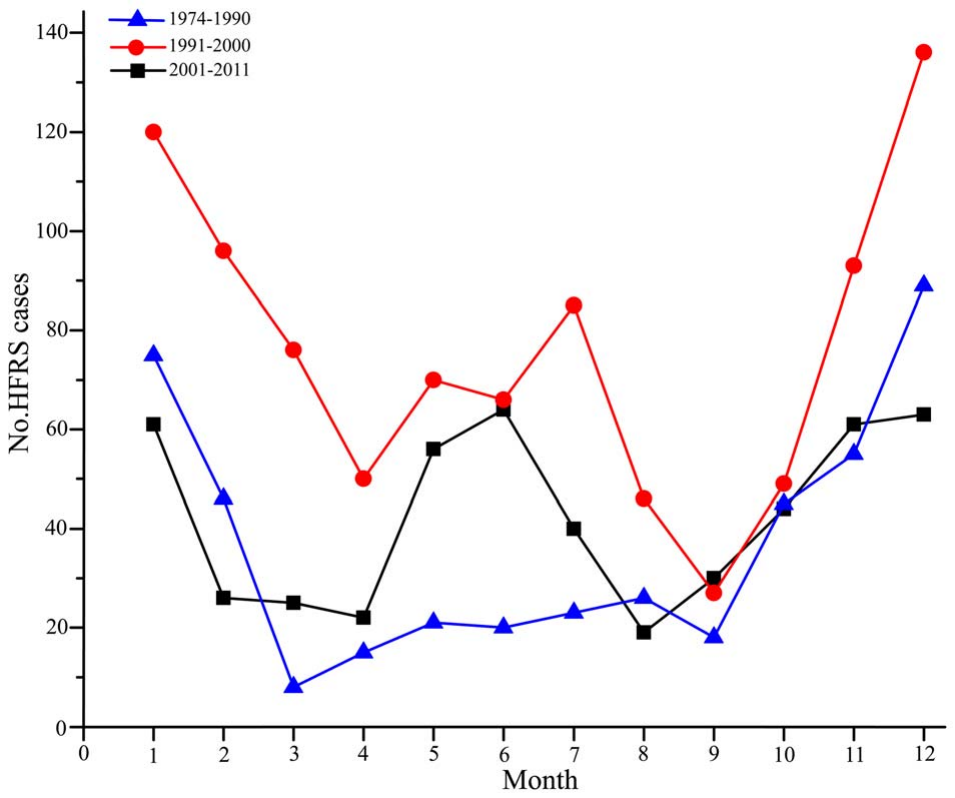
The monthly distribution (seasonality) of HFRS cases in Longquan. The seasonal distribution of HFRS cases during the period 1974–2011 in Longquan city.

The seasonality of HFRS noted above may provide important clues to its cause [9]. We therefore analyzed the seasonality of HFRS in Longquan for different time periods during 1974–2011. HFRS cases occurred in winter (November to January) and in spring/summer (May to July) at respective frequencies of 49.66% and 14.51% during 1974–1990, 38.18% and 24.18% in 1991–2000, and 36.20% and 31.31% in 2001–2011 (Figure 2). As the peak of HFRS associated with rats occurred in the spring, whereas HFRS associated with mice occurred mainly in the winter [9], the recent increase in cases in spring/summer suggests a rising disease toll due to rat-associated hantavirus(es) in Longquan. A similar seasonal shift, from mice-dominated to rat-dominated transmission, has been reported in other HFRS endemic regions [27].

### Hantavirus infection in small mammals

To analyze genetic diversity in the natural hantavirus reservoir and its relationship to those viruses found in humans, a total of 2,652 small mammals, representing 10 species of rodents and 3 species of insectivores (Table 1), were captured in Longquan during 2008–2011. *A. agrarius* mice and *M. fortis* voles were the dominant field species, accounting for 41.82% (1109) and 18.17% (482) of all small mammals collected, respectively. However, in residential areas the dominant species were rats of the family *Rattus* including 425 *R. losea* (16.02%), 372 *R. norvegicus* (14.03%), and 201 *R. flavipectus* (7.58%). Using IFA and RT-PCR, hantavirus antigens were detected in a total of 118 rodents including 78 *A. agrarius* (7.03%), 5 *M. fortis* (1.04%), 32 *R. norvegicus* (8.60%), and 3 *R. flavipectus* (1.49%). No hantaviruses were found in insectivores. Thus, the etiologic agents of HFRS cases in Longquan were likely hantaviruses carried by *A. agrarius* mice and *R. norvegicus* rats.

**Table 1:** Prevalence of hantavirus infection in small mammals in Longquan city, Zhejiang Province, China.

Species	2008		2009		2010		2011		Total
	Fields	Residential areas	Fields	Residential areas	Fields	Residential areas	Fields	Residential areas	
*Apodemus agrarius*	322/19	0/0	351/34	0/0	209/11	0/0	227/14	0/0	1109/78
*Crocidura attenuata*	0/0	0/0	3/0	0/0	0/0	0/0	0/0	0/0	3/0
*Microtus fortis*	135/1	0/0	114/4	0/0	91/0	0/0	142/0	0/0	482/5
*Mogera robusta*	0/0	0/0	2/0	0/0	0/0	0/0	0/0	0/0	2/0
*Mus musculus*	0/0	18/0	0/0	12/0	1/0	3/0	0/0	6/0	40/0
*Neomys fodiens*	0/0	0/0	1/0	0/0	1/0	0/0	0/0	0/0	2/0
*Niviventer confucianus*	0/0	0/0	4/0	0/0	4/0	0/0	1/0	0/0	9/0
*N. fulvescens*	0/0	0/0	1/0	0/0	0/0	0/0	4/0	0/0	5/0
*Rattus flavipectus*	157/0	0/0	0/0	1/0	0/0	0/0	0/0	43/3	201/3
*R. losea*	0/0	0/0	211/0	0/0	72/0	0/0	142/0	0/0	425/0
*R. nitidus*	0/0	0/0	0/0	0/0	0/0	0/0	1/0	0/0	1/0
*R. norvegicus*	13/0	59/4	3/0	46/5	3/0	9/0	4/0	235/23	372/32
*Sciurus igniventris*	0/0	0/0	0/0	0/0	0/0	0/0	1/0	0/0	1/0
Trap rate	7.00%	2.96%	6.61%	2.07%	6.50%	2.00%	6.61%	1.74%	3.99%
Total	627/20	77/4	690/38	59/5	381/11	12/0	522/14	284/26	2652/118

Note: Small mammals trapped/IFA and PCR positive.

### Serologic and phylogenetic analyses

Serum samples from five human patients were collected on day 1 of hospitalization. Samples were tested for IgM and IgG antibodies by IFA using GOUV- or HTNV-infected cells (Table S1). Three serum samples showed higher IgM and IgG titers in HTNV- specific IFA, and one in GOUV-specific IFA. One sample showed higher IgM titers in HTNV-specific IFA, but with the same titers in HTNV- or GOUV specific IFA, suggesting cross-reactivity of HTNV with GOUV.

To further characterize the etiologic agents of human infection in Longquan, complete or partial hantavirus M segment sequences were recovered from 118 hantavirus antigen-positive rodent samples (Table S2). In addition, complete S segment sequences were amplified from all 118 hantavirus antigen-positive rodent lung tissues, and partial S segment sequences were recovered from the five human serum samples collected from patients with acute HFRS.

**Figure 3:**
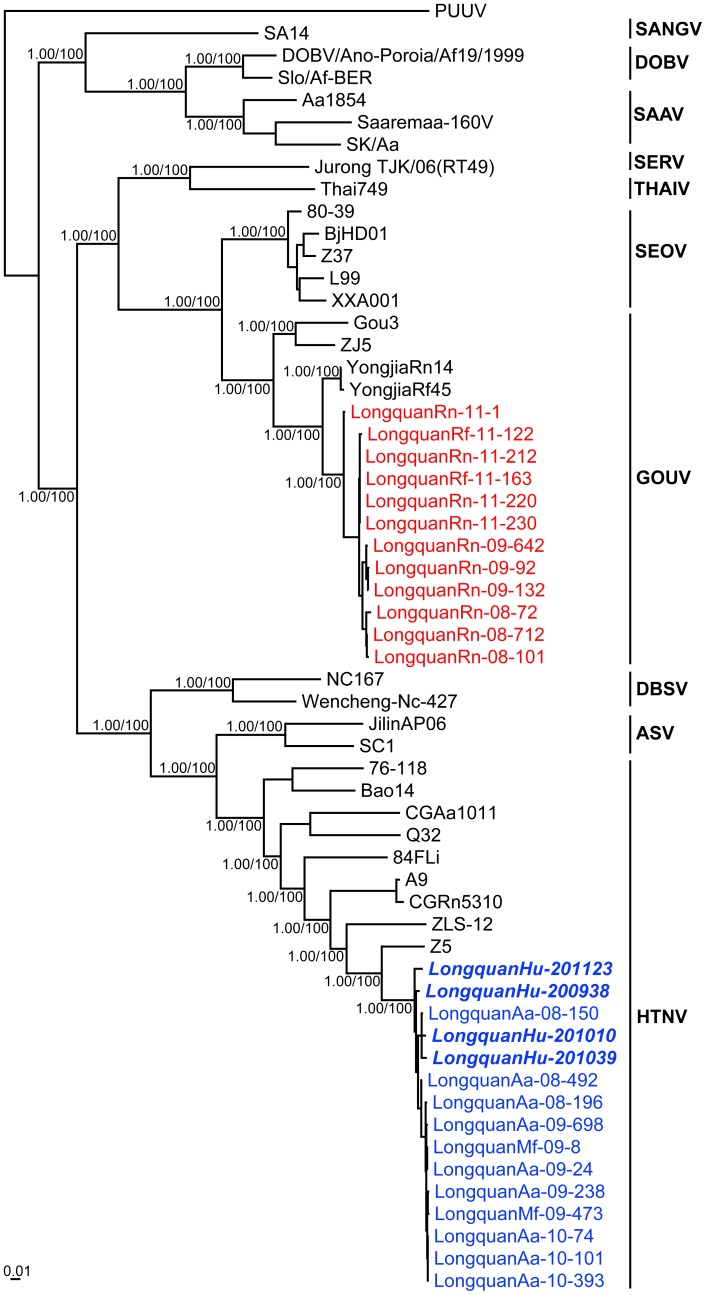
Phylogenetic relationships of hantaviruses identified in Longquan based on partial M segment sequences. Sequences obtained in this study are shown in blue (HTNV) and red (GOUV), with those viruses collected from humans shown in italics. Numbers (>0.7/>70%) at nodes indicate posterior probabilities and bootstrap support values. Puumala virus (PUUV) was used as an outgroup. All GenBank accession numbers are described in Table S2. The scale bar represents the number of nucleotide substitutions per site.

**Figure 4:**
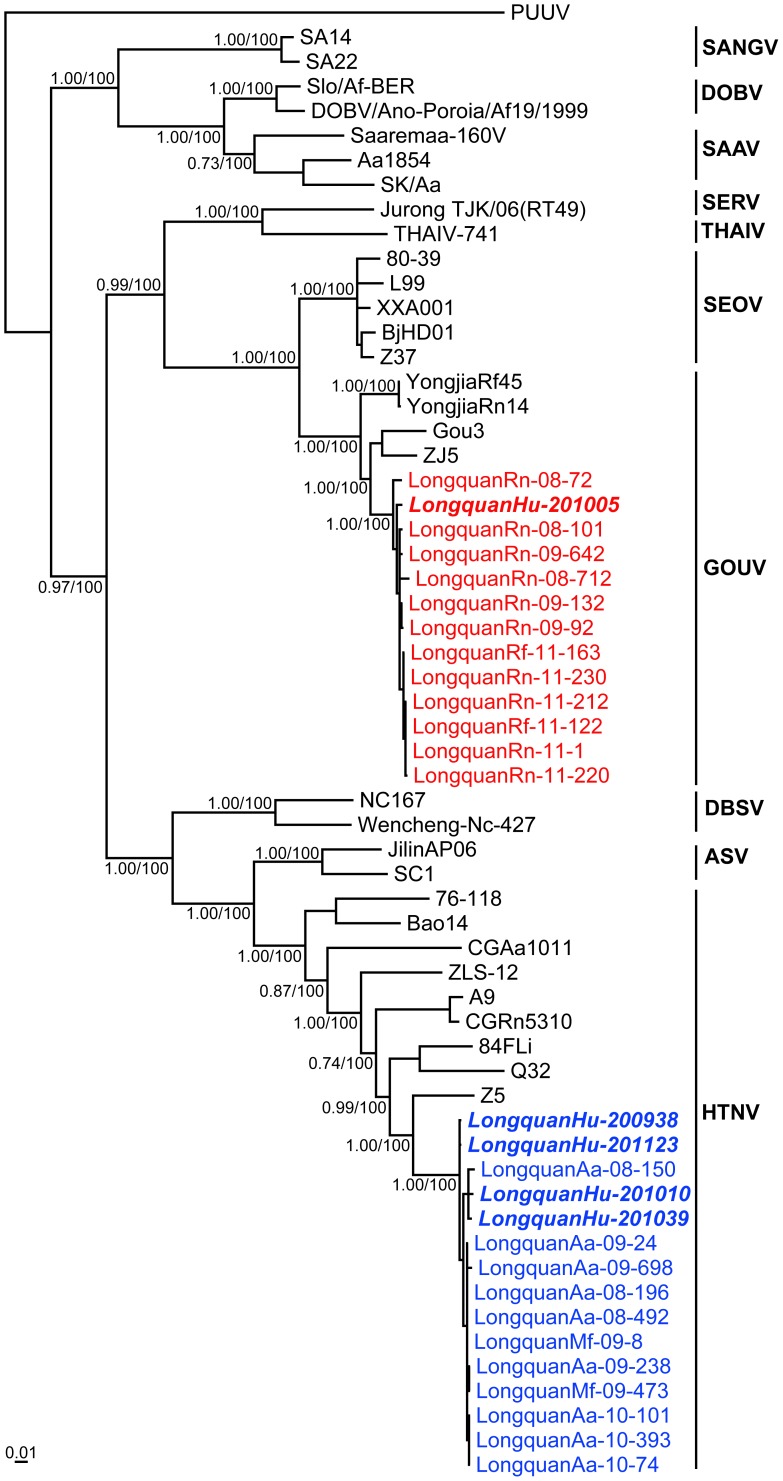
Phylogenetic relationships of hantaviruses identified in Longquan based on partial S segment sequences. Sequences obtained in this study are shown in blue (HTNV) and red (GOUV), with those viruses collected from humans shown in italics. Numbers (>0.7/>70%) at nodes indicate posterior probabilities and bootstrap values. Puumala virus (PUUV) was used as an outgroup. All GenBank accession numbers are given in Table S2. The scale bar represents the number of nucleotide substitutions per site.

Notably, the sequences recovered from 78 *A. agrarius*, 5 *M. fortis*, and 4 human samples were very closely related to each other, with 98.2–100% nt and 98.4–100% aa sequence identities in the M segment and 98.6–100%/99.3–100% identities in the S segment. This similarity is indicative of direct viral transmission from rodents to humans. To determine the phylogenetic relationships among the viruses described here and to known hantaviruses, phylogenetic trees were estimating using the M and S segment sequences (in which Bayesian and ML methods produced similar topologies). Most notably, the sequences sampled from *A. agrarius* mice, *M. fortis* voles, and humans clustered together and formed a distinct and well-supported lineage in both trees (Figures 3–4). Interestingly, these strains also exhibited a close evolutionary relationship to strains HTNV and Z5 previously isolated from *A. agrarius* mice in Zhejiang Province [28].

All hantavirus sequences recovered from rats were very closely related to each other, with 97.5–100% nt and 98.5–100% aa sequence identities in the M segment and 98.5–100%/98.6–100% identities in the S segment. The partial S segment sequence recovered from the serum of a human patient in which high titers of IgG antibodies against GOUV had been detected (Table S1) was very closely related to sequences recovered from rats (98.9–99.5%/99.2–100%). Remarkably, these hantavirus sequences were closely related to previously described variants of GOUV – Gou3, ZJ5, YongjiaRf45 and YongjiaRn14 – but more distant from variants of SEOV. In phylogenetic trees of the M or S segments those strains from *Rattus* rats (*R. flavipectus* and *R. norvegicus*) and human clustered together, forming a distinct and strongly supported cluster (posterior probabilities of 1.0 for both M and S sequences) within the broader group of GOUV sequences (Figures 3–4). This phylogenetic pattern is indicative of a new genetic variant of GOUV in Longquan.

## Discussion

HFRS was a serious problem in China during the 1980 and 1990s [7], [9]. As a result of comprehensive preventive measures and improved living conditions, the incidence of HFRS in China has declined dramatically during the last decade [7]. Because of favorable ecological conditions and low socioeconomic status in rural areas, farmers have frequently been the major victims of HFRS, both inside and outside of China [5], [7], [9], [29]–[31]. The annual number of registered HFRS cases in Longquan has decreased, from 138 in 1998 to 46 in 2011, with a similar pattern observed in other parts of China [7], [32]. However, the incidence rate (>10 cases/100,000 population) in this region is still the highest in China despite ongoing vaccination. Considering the dramatic decrease in the rural population of Longquan in recent years (at least 30% of the rural population had moved into cities or towns by the end of 1990s), the real incidence rate of HFRS in rural areas may be much higher than reported here. In addition, the prevalence of hantavirus infection is high in rodents in both rural and residential areas in Longquan, especially GOUV in Norway rats (>8%). Thus, hantavirus infection will likely remain a major public health problem for the foreseeable future in Longquan city.

GOUV was first isolated from *R. rattus* captured in Zhejiang Province in 2000 [11], and initially considered as a variant of SEOV [10], [11]. However, GOUV is distinct from SEOV both serologically and genetically [11] and is found in a different rat species (*R. rattus*, *R. flavipectus*). Such distinction means that GOUV is currently defined as a tentative hantavirus species by the International Committee on Taxomony of Viruses (ICTV) [14]. In this study hantavirus variants originating from rats (*R. flavipectus* and *R. norvegicus*) from Longquan were most closely related to GOUV, forming a distinct and strongly supported lineage in both the M and S segment trees. Hence, these data suggest that the hantavirus variants carried by *Rattus* rats in Longquan represent a new genetic variant of GOUV. As no SEOV or other hantaviruses have been found in *Rattus* rats from Longquan and the sequences recovered from one patient in Longquan belonged to GOUV, our data clearly indicate that GOUV carried by *Rattus* rats (*R. flavipectus* and *R. norvegicus*) can cause human disease, and that there is ongoing spillover from the rodent reservoir to the human population. This is the first report of GOUV being associated with human HFRS cases since its discovery in 2000 [11]. Accordingly, further studies are needed to determine the pathogenicity and severity of GOUV in humans, as well as the possibility of human-to-human transmission.

Similar to other hantaviruses [2], [33], HTNV exhibits considerable genetic diversity and displays a geographic clustering of genetic variants, especially in mountainous regions [34]. To date, at least nine genetic lineages of HTNV have been found in *Apodemus* mice in Eastern Asia [34]. In this study, the virus sequences recovered from *Apodemus* mice and *Microtus* voles in Longquan formed a distinct lineage within HTNV in both the M and S segment trees, suggesting that a new genetic variant of HTNV is circulating in Longquan. Our earlier studies in the northeast and central parts of China documented Yuanjiang virus (YUJV) and Vladivostok virus (VLAV) in *M. fortis* voles, respectively [35]. However, these viruses were not detected in the *Microtus* voles from Longquan. Additional study is needed to determine if these viruses are present and whether they are pathogenic to humans in the HFRS-affected region.

Previous investigations revealed that HFRS caused by HTNV transmitted by *Apodemus* mice occurred mainly in winter, while the peak of HFRS caused by hantaviruse(s) transmitted by *Rattus* rats was in spring [9], [36], [37]. The seasonal analyses of HFRS cases performed here indicated that most of the HFRS cases registered in Longquan during 1974–1998 occurred in winter, in turn suggesting that human infections were due to HTNV. However, during the last decade the peak of HFRS has shifted to the spring in Longquan, with a similar pattern observed in other HFRS endemic regions [27]. In addition, GOUV was highly prevalent in *R. norvegicus* in residential areas, and more so than HNTV in *Apodemus* mice (prevalences of 8.60% and 7.03%, respectively). In sum, these data suggest that hantavirus(es) carried by rats may have become the major cause of HFRS in Longquan city over the past decade.

In conclusion, we have shown that hantavirus infection is endemic in both humans and rodents in Longquan, with the latter acting as a major reservoir for the former. Epidemiological and phylogenetic analyses indicate that GOUV and HTNV are circulating in local rodents and have a direct connection to the human population. As rats (*Rattus* species) are more mobile than the hosts of other hantaviruses [10], this study strongly reinforces the need for vigilance in preventing the spillover of GOUV from rats in China.

## Supporting Information

Checklist S1 STROBE checklist.
(DOC)

Table S1 Serological and RT-PCR assay of five serum samples collected from HFRS patients in Longquan city, China.
(DOC)

Table S2 Hantavirus strains obtained in this study and those taken from GenBank.
(DOC)

## References

[1] Meyer BJ, Schmaljohn CS (2000) Persistent hantavirus infections: characteristics and mechanisms. Trends Microbiol 8: 61–67. 10664598 10.1016/s0966-842x(99)01658-3

[2] Jonsson CB, Figueiredo LT, Vapalahti O (2010) A global perspective on hantavirus ecology, epidemiology, and disease. Clin Microbiol Rev 23: 412–441. 20375360 10.1128/CMR.00062-09 PMC2863364

[3] Watson DC, Sargianou M, Papa A, Chra P, Starakis I, et al (2013) Epidemiology of Hantavirus infections in humans: A comprehensive, global overview. Crit Rev Microbiol [epub ahead of print]. 10.3109/1040841X.2013.783555 23607444

[4] Gledovic ZB, Jeknic AS, Grgurevic AD, Rakocevic BB, Bozovic BR, et al (2008) Hemorrhagic fever with renal syndrome in Montenegro. Jpn J Infect Dis 61: 386–387. 18806348

[5] Vapalahti O, Mustonen J, Lundkvist A, Henttonen H, Plyusnin A, et al (2003) Hantavirus infections in Europe. Lancet Infect Dis 3: 653–661. 14522264 10.1016/s1473-3099(03)00774-6

[6] Zhang X, Chen HY, Zhu LY, Zeng LL, Wang F, et al (2011) Comparison of Hantaan and Seoul viral infections among patients with hemorrhagic fever with renal syndrome (HFRS) in Heilongjiang, China. Scand J Infect Dis 43: 632–641. 21428852 10.3109/00365548.2011.566279

[7] Zhang YZ, Zou Y, Fu ZF, Plyusnin A (2010) Hantavirus infections in humans and animals, China. Emerg Infect Dis 16: 1195–1203. 20678311 10.3201/eid1608.090470 PMC3298307

[8] Hjertqvist M, Klein SL, Ahlm C, Klingstrom J (2010) Mortality rate patterns for hemorrhagic fever with renal syndrome caused by Puumala virus. Emerg Infect Dis 16: 1584–1586. 20875284 10.3201/eid1610.100242 PMC3294390

[9] Chen HX, Qiu FX, Dong BJ, Ji SZ, Li YT, et al (1986) Epidemiological studies on hemorrhagic fever with renal syndrome in China. J Infect Dis 154: 394–398. 2874178 10.1093/infdis/154.3.394

[10] Lin XD, Guo WP, Wang W, Zou Y, Hao ZY, et al (2012) Migration of norway rats resulted in the worldwide distribution of Seoul hantavirus today. J Virol 86: 972–981. 22090114 10.1128/JVI.00725-11 PMC3255798

[11] Wang H, Yoshimatsu K, Ebihara H, Ogino M, Araki K, et al (2000) Genetic diversity of hantaviruses isolated in china and characterization of novel hantaviruses isolated from *Niviventer confucianus* and *Rattus rattus* . Virology 278: 332–345. 11118357 10.1006/viro.2000.0630

[12] Lin XD, Wang W, Guo WP, Zhang XH, Xing JG, et al (2012) Cross-species transmission in the speciation of the currently known *Murinae*-associated hantaviruses. J Virol 86: 11171–11182. 22855492 10.1128/JVI.00021-12 PMC3457156

[13] Guo WP, Lin XD, Wang W, Zhang XH, Chen Y, et al (2011) A new subtype of Thottapalayam virus carried by the Asian house shrew (*Suncus murinus*) in China. Infect Genet Evol 11: 1862–1867. 21801855 10.1016/j.meegid.2011.07.013

[14] Plyusnin A, Beaty BJ, Elliott RM, Goldbach R, Kormelink R, et al.. (2012) Bunyaviridae. In King AMQ, Lefkowitz EJ, Adams MJ, Karstens EB, editors. Virus Taxonomy: Classification and nomenclature of viruses: Ninth Report of the International Committee on Taxonomy of Viruses. San Diego: Elsevier. pp. 693–709.

[15] Guo WP, Lin XD, Wang W, Tian JH, Cong ML, et al (2013) Phylogeny and origins of hantaviruses harbored by bats, insectivores, and rodents. PLoS Pathog 9: e1003159. 23408889 10.1371/journal.ppat.1003159 PMC3567184

[16] Wang MR, Wang W, Lin XD, Mei SH, Guo WP, et al (2011) Investigation on the natural infectious status of hantaviruses among small mammals in Longquan city, Zhejiang province [in Chinese]. Zhonghua liu xing bing xue za zhi 32: 598–601. 21781480

[17] Mills JN, Childs JE, Ksiazek TG, Peters CJ, Velleca WM (1995) Methods for trapping and sampling small mammals for virologic testing. Atlanta: Centers for Disease Control and Prevention.

[18] Zhang YZ, Dong X, Li X, Ma C, Xiong HP, et al (2009) Seoul virus and hantavirus disease, Shenyang, People's Republic of China. Emerg Infect Dis 15: 200–206. 19193263 10.3201/eid1502.080291 PMC2662651

[19] Schmaljohn CS, Jennings GB, Hay J, Dalrymple JM (1986) Coding strategy of the S genome segment of Hantaan virus. Virology 155: 633–643. 3024404 10.1016/0042-6822(86)90223-0

[20] Zou Y, Hu J, Wang ZX, Wang DM, Yu C, et al (2008) Genetic characterization of hantaviruses isolated from Guizhou, China: evidence for spillover and reassortment in nature. J Med Virol 80: 1033–1041. 18428127 10.1002/jmv.21149

[21] Zhang YZ, Zhang FX, Wang JB, Zhao ZW, Li MH, et al (2009) Hantaviruses in rodents and humans, Inner Mongolia Autonomous Region, China. Emerg Infect Dis 15: 885–891. 19523286 10.3201/eid1506.081126 PMC2727351

[22] Klempa B, Fichet-Calvet E, Lecompte E, Auste B, Aniskin V, et al (2007) Novel hantavirus sequences in Shrew, Guinea. Emerg Infect Dis 13: 520–522. 10.3201/eid1303.061198 PMC2725914 17554814

[23] Ronquist F, Huelsenbeck JP (2003) MrBayes 3: Bayesian phylogenetic inference under mixed models. Bioinformatics 19: 1572–1574. 12912839 10.1093/bioinformatics/btg180

[24] Stamatakis A, Hoover P, Rougemont J (2008) A rapid bootstrap algorithm for the RAxML Web servers. Syst Biol 57: 758–771. 18853362 10.1080/10635150802429642

[25] Posada D (2008) jModelTest: phylogenetic model averaging. Mol Biol Evol 25: 1253–1256. 18397919 10.1093/molbev/msn083

[26] Page RD (1996) TreeView: an application to display phylogenetic trees on personal computers. Comput Appl Biosci 12: 357–358. 8902363 10.1093/bioinformatics/12.4.357

[27] Fang LQ, Wang XJ, Liang S, Li YL, Song SX, et al (2010) Spatiotemporal trends and climatic factors of hemorrhagic fever with renal syndrome epidemic in Shandong Province, China. PLoS Negl Trop Dis 4: e789. 20706629 10.1371/journal.pntd.0000789 PMC2919379

[28] Li C, Xie RH, Zhu HP, Xu F, Yao PP, et al (2010) Full-length nucleotide sequence analysis of the S and M segments in Z5 strain of Hantavirus [in Chinese]. Chin J Zoonoses 26: 215–217.

[29] Schmaljohn C, Hjelle B (1997) Hantaviruses: a global disease problem. Emerg Infect Dis 3: 95–104. 9204290 10.3201/eid0302.970202 PMC2627612

[30] Chen HX, Qiu FX (1993) Epidemiologic surveillance on the hemorrhagic fever with renal syndrome in China. Chin Med J (Engl) 106: 857–863. 7908258

[31] Bi P, Tong S, Donald K, Parton K, Ni J (2002) Climatic, reservoir and occupational variables and the transmission of haemorrhagic fever with renal syndrome in China. Int J Epidemiol 31: 189–193. 11914320 10.1093/ije/31.1.189

[32] Kang YJ, Zhou DJ, Tian JH, Yu B, Guo WP, et al (2012) Dynamics of hantavirus infections in humans and animals in Wuhan city, Hubei, China. Infect Genet Evol 12: 1614–1621. 22910184 10.1016/j.meegid.2012.07.017

[33] Sironen T, Vaheri A, Plyusnin A (2001) Molecular evolution of Puumala hantavirus. J Virol 75: 11803–11810. 11689661 10.1128/JVI.75.23.11803-11810.2001 PMC114766

[34] Zou Y, Hu J, Wang ZX, Wang DM, Li MH, et al (2008) Molecular diversity and phylogeny of Hantaan virus in Guizhou, China: evidence for Guizhou as a radiation center of the present Hantaan virus. J Gen Virol 89: 1987–1997. 18632971 10.1099/vir.0.2008/000497-0

[35] Zou Y, Xiao QY, Dong X, Lv W, Zhang SP, et al (2008) Genetic analysis of hantaviruses carried by reed voles *Microtus fortis* in China. Virus Res 137: 122–128. 18644410 10.1016/j.virusres.2008.06.012

[36] Li YL, Ruo SL, Tong Z, Ma QR, Liu ZL, et al (1995) A serotypic study of hemorrhagic fever with renal syndrome in rural China. Am J Trop Med Hyg 52: 247–251. 7694967 10.4269/ajtmh.1995.52.247

[37] Zhang YZ, Lin XD, Shi NF, Wang W, Liao XW, et al (2010) Hantaviruses in small mammals and humans in the coastal region of Zhejiang Province, China. J Med Virol 82: 987–995. 20419812 10.1002/jmv.21737

