# Supplementary material for: Ongoing Spillover of Hantaan and Gou Hantaviruses from Rodents Is Associated with Hemorrhagic Fever with Renal Syndrome (HFRS) in China
Source: PLoS Negl Trop Dis. 2013 Oct 17;7(10):e2484. doi: 10.1371/journal.pntd.0002484 (PMC3798614; doi:10.1371/journal.pntd.0002484)
Supplement: Table S1 — Serological and RT-PCR assay of five serum samples collected from HFRS patients in Longquan city, China. (DOC) [file pntd.0002484.s002.doc]

Table S1. Serological and RT-PCR assay of five serum samples collected from HFRS patients in Longquan city, China.

| Serum sample | IgM assay | | IgG assay | | PCR |
| --- | --- | --- | --- | --- | --- |
|  | HTNV | GOUV | HTNV | GOUV |  |
| LongquanHu-200938 | 640 | 640 | 2560 | 1280 | HTNV |
| LongquanHu-201005 | - | 20 | 320 | 640 | GOUV |
| LongquanHu-201010 | 160 | 160 | 2560 | 1280 | HTNV |
| LongquanHu-201038 | 80 | 40 | 640 | 640 | HTNV |
| LongquanHu-201123 | 320 | 160 | 2580 | 1280 | HTNV |
