# Supplementary material for: Ongoing Spillover of Hantaan and Gou Hantaviruses from Rodents Is Associated with Hemorrhagic Fever with Renal Syndrome (HFRS) in China
Source: PLoS Negl Trop Dis. 2013 Oct 17;7(10):e2484. doi: 10.1371/journal.pntd.0002484 (PMC3798614; doi:10.1371/journal.pntd.0002484)
Supplement: Table S2 — Hantavirus strains obtained in this study and those taken from GenBank. (DOC) [file pntd.0002484.s003.doc]

Table S2. Hantavirus strains obtained in this study and those taken from GenBank.

| **Virus** | **GenBank accession number** | |
| --- | --- | --- |
|  | **S** | **M** |
| LongquanAa-08-150 | JQ912697 | JQ912803 |
| LongquanAa-08-157 | JQ912698 | JQ912804 |
| LongquanAa-08-196 | JQ912699 | JQ912805 |
| LongquanAa-08-200 | JQ912700 | JQ912806 (partial) |
| LongquanAa-08-218 | JQ912701 | JQ912807 (partial) |
| LongquanAa-08-221 | JQ912702 | JQ912808 (partial) |
| LongquanAa-08-224 | JQ912703 | JQ912809 (partial) |
| LongquanAa-08-235 | JQ912704 | JQ912810 |
| LongquanAa-08-272 | JQ912705 | JQ912811 (partial) |
| LongquanAa-08-278 | JQ912706 | JQ912812 |
| LongquanAa-08-284 | JQ912707 | JQ912813 (partial) |
| LongquanAa-08-300 | JQ912708 | JQ912814 |
| LongquanAa-08-314 | JQ912709 | JQ912815 (partial) |
| LongquanAa-08-358 | JQ912710 | JQ912816 (partial) |
| LongquanAa-08-481 | JQ912711 | JQ912817 (partial) |
| LongquanAa-08-492 | JQ912712 | JQ912818 |
| LongquanAa-08-542 | JQ912713 | JQ912819 (partial) |
| LongquanAa-08-573 | JQ912714 | JQ912820 (partial) |
| LongquanAa-08-595 | JQ912715 | JQ912821 (partial) |
| LongquanAa-09-113 | JQ912716 | JQ912822 (partial) |
| LongquanAa-09-14 | JQ912717 | JQ912823 (partial) |
| LongquanAa-09-171 | JQ912718 | JQ912824 |
| LongquanAa-09-191 | JQ912719 | JQ912825 |
| LongquanAa-09-192 | JQ912720 | JQ912826 (partial) |
| LongquanAa-09-233 | JQ912721 | JQ912827 (partial) |
| LongquanAa-09-238 | JQ912722 | JQ912828 |
| LongquanAa-09-24 | JQ912723 | JQ912829 |
| LongquanAa-09-27 | JQ912724 | JQ912830 (partial) |
| LongquanAa-09-304 | JQ912725 | JQ912831 (partial) |
| LongquanAa-09-329 | JQ912726 | JQ912832 (partial) |
| LongquanAa09342 | JQ912727 | JQ912833 (partial) |
| LongquanAa-09-344 | JQ912728 | JQ912834 (partial) |
| LongquanAa-09-350 | JQ912729 | JQ912835 |
| LongquanAa-09-355 | JQ912730 | JQ912836 (partial) |
| LongquanAa-09-383 | JQ912731 | JQ912837 (partial) |
| LongquanAa-09-406 | JQ912732 | JQ912838 (partial) |
| LongquanAa-09-41 | JQ912733 | JQ912839 (partial) |
| LongquanAa-09-44 | JQ912734 | JQ912840 (partial) |
| LongquanAa-09-458 | JQ912735 | JQ912841 (partial) |
| LongquanAa-09-475 | JQ912736 | JQ912842 (partial) |
| LongquanAa-09-476 | JQ912737 | JQ912843 |
| LongquanAa-09-489 | JQ912738 | JQ912844 |
| LongquanAa-09-490 | JQ912739 | JQ912845 (partial) |
| LongquanAa-09-518 | JQ912740 | JQ912846 (partial) |
| LongquanAa-09-52 | JQ912741 | JQ912847 (partial) |
| LongquanAa-09-536 | JQ912742 | JQ912848 (partial) |
| LongquanAa-09-538 | JQ912743 | JQ912849 (partial) |
| LongquanAa-09-541 | JQ912744 | JQ912850 |
| LongquanAa-09-626 | JQ912745 | JQ912851 |
| LongquanAa-09-689 | JQ912746 | JQ912852 |
| LongquanAa-09-697 | JQ912747 | JQ912853 (partial) |
| LongquanAa-09-698 | JQ912748 | JQ912854 |
| LongquanAa-09-98 | JQ912749 | JQ912855 (partial) |
| LongquanAa-10-101 | JQ912750 | JQ912856 |
| LongquanAa-10-116 | JQ912751 | JQ912857 (partial) |
| LongquanAa-10-19 | JQ912752 | JQ912858 (partial) |
| LongquanAa-10-393 | JQ912753 | JQ912859 |
| LongquanAa-10-46 | JQ912754 | JQ912860 (partial) |
| LongquanAa-10-58 | JQ912755 | JQ912861 |
| LongquanAa-10-64 | JQ912756 | JQ912862 (partial) |
| LongquanAa-10-68 | JQ912757 | JQ912863 (partial) |
| LongquanAa-10-74 | JQ912758 | JQ912864 |
| LongquanAa-10-85 | JQ912759 | JQ912865 (partial) |
| LongquanAa-10-98 | JQ912760 | JQ912866 |
| LongquanAa-11-398 | KC344241 | KC344256 (partial) |
| LongquanAa-11-405 | KC344240 | KC344255 (partial) |
| LongquanAa-11-414 | KC344239 | KC344254 (partial) |
| LongquanAa-11-452 | KC344236 | KC344264 |
| LongquanAa-11-473 | KC344249 | KC344263 |
| LongquanAa-11-619 | KC344248 | KC344253 (partial) |
| LongquanAa-11-641 | KC344238 | KC344266 (partial) |
| LongquanAa-11-642 | KC344245 | KC344262 |
| LongquanAa-11-694 | KC344247 | KC344261 |
| LongquanAa-11-701 | KC344244 | KC344265 (partial) |
| LongquanAa-11-744 | KC344243 | KC344260 |
| LongquanAa-11-745 | KC344237 | KC344259 |
| LongquanAa-11-754 | KC344246 | KC344258 |
| LongquanAa-11-774 | KC344242 | KC344257 |
| LongquanHu-200938 | JQ912761 (partial) | JQ912867 (partial) |
| LongquanHu-201005 | JQ912762 (partial) | - |
| LongquanHu-201010 | JQ912763 (partial) | JQ912868 (partial) |
| LongquanHu-201039 | JQ912764 (partial) | JQ912869 (partial) |
| LongquanHu-201123 | JQ912765 (partial) | JQ912870 (partial) |
| LongquanMf-08-92 | JQ912766 | JQ912871 (partial) |
| LongquanMf-09-189 | JQ912767 | JQ912872 (partial) |
| LongquanMf-09-473 | JQ912768 | JQ912873 |
| LongquanMf-09-556 | JQ912769 | JQ912874 (partial) |
| LongquanMf-09-8 | JQ912770 | JQ912875 |
| LongquanRf-11-122 | JQ912771 | JQ912876 |
| LongquanRf-11-163 | JQ912772 | JQ912877 |
| LongquanRf-11-372 | KC344252 | KC344269 |
| LongquanRn-08-101 | JQ912773 | JQ912878 |
| LongquanRn-08-242 | JQ912774 | JQ912879 |
| LongquanRn-08-712 | JQ912775 | JQ912880 |
| LongquanRn-08-72 | JQ912776 | JQ912881 |
| LongquanRn-09-132 | JQ912777 | JQ912882 |
| LongquanRn-09-374 | JQ912778 | JQ912883 (partial) |
| LongquanRn-09-641 | JQ912779 | JQ912884 (partial) |
| LongquanRn-09-642 | JQ912780 | JQ912885 |
| LongquanRn-09-92 | JQ912781 | JQ912886 |
| LongquanRn-11-1 | JQ912782 | JQ912887 |
| LongquanRn-11-104 | JQ912783 | JQ912888 |
| LongquanRn-11-11 | JQ912784 | JQ912889 |
| LongquanRn-11-12 | JQ912785 | JQ912890 |
| LongquanRn-11-123 | JQ912786 | JQ912891 |
| LongquanRn-11-127 | JQ912787 | JQ912892 |
| LongquanRn-11-128 | JQ912788 | JQ912893 |
| LongquanRn-11-148 | JQ912789 | JQ912894 |
| LongquanRn-11-18 | JQ912790 | JQ912895 |
| LongquanRn-11-20 | JQ912791 | JQ912896 |
| LongquanRn-11-212 | JQ912792 | JQ912897 |
| LongquanRn-11-214 | JQ912793 | JQ912898 |
| LongquanRn-11-218 | JQ912794 | JQ912899 |
| LongquanRn-11-220 | JQ912795 | JQ912900 |
| LongquanRn-11-230 | JQ912796 | JQ912901 |
| LongquanRn-11-234 | JQ912797 | JQ912902 |
| LongquanRn-11-239 | JQ912798 | JQ912903 |
| LongquanRn-11-296 | KC344250 | KC344267 |
| LongquanRn-11-3 | JQ912799 | JQ912904 |
| LongquanRn-11-300 | JQ912800 | JQ912905 |
| LongquanRn-11-332 | KC344251 | KC344268 |
| LongquanRn-11-4 | JQ912801 | JQ912906 |
| LongquanRn-11-99 | JQ912802 | JQ912907 |
|  |  |  |
| PUUV | NC_005224 | X61034 |
| SA14 | JQ082300 | JQ082301 |
| SA22 | JQ082303 | - |
| DOBV/Ano-Poroia/Af19/1999 | NC_005233 | NC_005234 |
| Slo/Af-BER | GU904029 | GU904035 |
| Aa1854 | EU188452 | EU188453 |
| Saaremaa-160V | AJ009773 | AJ009774 |
| SK/Aa | AY961615 | AY961616 |
| Jurong TJK/06(RT49) | GQ274940 | GQ27493 |
| Thai749 | - | L08756 |
| THAIV-741 | AB186420 | - |
| 80-39 | AY273791 | S47716 |
| BjHD01 | AY627049 | DQ133505 |
| Z37 | AF187082 | AF190119 |
| L99 | AF288299 | AF288298 |
| XXA001 | EF210133 | EF210127 |
| Gou3 | AF184988 | AB027512 |
| ZJ5 | FJ753400 | FJ811839 |
| YongjiaRn14 | GU592947 | GU592927 |
| YonjiaRf45 | GU592943 | GU592926 |
| NC167 | AB027523 | AB027115 |
| Wencheng-Nc-427 | JF796017 | JF796031 |
| JilinAP06 | EF121324 | EF371454 |
| SC1 | AY675349 | AY675353 |
| 76-118 | M14626 | M14627 |
| Bao14 | AB127998 | AB127995 |
| CGAa1011 | EF990913 | EF990927 |
| Q32 | AB027097 | DQ371905 |
| 84FLi | AY017064 | AF345636 |
| A9 | AF329390 | AF035831 |
| CGRn5310 | EF990906 | EF990920 |
| ZLS-12 | FJ753398 | FJ753399 |
| Z5 | FJ753400 | EU074224 |
